# Miguel Llinás and the Structure of the Kringle Fold

**DOI:** 10.1007/s10930-021-09981-w

**Published:** 2021-03-31

**Authors:** Laszlo Patthy

**Affiliations:** Institute of Enzymology, Research Centre for Natural Sciences, 1117 Budapest, Hungary

The mid-1970s and early 1980s have witnessed a burst of interest in fibrinolytic proteases as it became clear, primarily through the pioneering work of Edward Reich and his coworkers (The Rockefeller University) that these enzymes might play an important role in tumor invasion and metastasis [1–5]. The observation that oncogenic transformation leads to loss of fibronectin and reduced adhesion of tumor cells, the hypothesis that loss of fibronectin is due to its proteolytic degradation, have further increased interest in the role of extracellular matrix proteins and proteases in tumor metastasis [6, 7].

The first major advances in the structural biology of fibrinolytic enzymes and fibronectin were made by the laboratory of Staffan Magnusson (University of Aarhus). Magnusson and coworkers have determined the primary sequence of plasminogen and have shown that five regions in the non-protease part of this protein show significant sequence homology with two internally homologous structures of prothrombin [8–10]. They have used the term kringle for these homologous regions since the two dimensional representation of their disulfide-bridged structures resemble the classical shape of this Scandinavian cake [8–10]. The Magnusson lab has also determined the primary sequence of fibronectin. This large protein was found to have three types of internal homology regions (type I, type II and type III repeats), indicating that a number of partial internal gene duplications have occurred during the evolution of this multidomain protein [11, 12].

Our research group was attracted to this field at the end of the 70s with a view of clarifying structure-function aspects of plasminogen, plasminogen activators and fibronectin, focusing on kringles of plasminogen and the three types of internal homology units of fibronectin. We have demonstrated that the kringle 5 domain of human plasminogen carries a benzamidine-binding site [13], but we focused primarily on kringles 1 and 4 that were known to be responsible for the lysine-affinity of plasminogen. Based on chemical modification studies, we have demonstrated that the primary determinants of the lysine-binding site of kringle 4 are Arginine 70 and Aspartic acid 56 that provide the positive and negative charges necessary for electrostatic binding of the ligand’s carboxylate and ammonium groups [14]. In the case of kringle 1 domain of plasminogen, we have shown that Arginines 32 and 34 are essential for the fibrin affinity of this domain [15]. Parallel with these structure-function studies, at the suggestion of Robert Williams (University of Oxford) we have started a collaboration to solve the solution structure of kringle 4 by NMR spectroscopy. Comparison of the NMR spectrum of kringle 4 with the spectra of various kringle 4 species chemically modified at defined positions has permitted the assignment of several resonances to specific residues in the kringle 4 sequence [16, 17]. The NOE studies on kringle 4 revealed that Leucine 45 is in close proximity of the sequentially distant Trp25/Trp61 residue pair, delineating a key structural feature of the kringle-fold. The binding of 6-aminohexanoic acid to kringle 4 was shown to cause shifts in the resonances of Trp71 (neighboring the ligand-binding Arginine 70), suggesting that it may be lining the ω-aminocarboxylic acid binding site of the kringle. This localization of the binding site was in harmony with the result of Hochschwender and Laursen that modification of Trp71 results in loss of ligand affinity of kringle 4 [18].

During the course of these studies we have become aware of the collaborative efforts of the lab of Richard Laursen (Boston University) and the lab of Miguel Llinás (Carnegie-Mellon University) to study the structure of plasminogen kringles by NMR spectroscopy [19–21]. In agreement with Robert Williams, we have decided to join forces, rather than duplicate efforts on kringle 4 [22]. We have participated in a collaboration with Miguel in a comparative study of human, porcine, bovine and chicken kringle 4 domains that has significantly facilitated resonance assignment [23–25]. Miguel continued his impressive NMR studies on kringle 1and kringle 4 of plasminogen with the Laursen lab [26–32], but he has also extended his studies to kringle 3 [33] and kringle 5 of plasminogen [34–37], the kringle 2 domain of the tissue-type plasminogen activator [38–41] and the kringle of urokinase [42, 43], making him an unquestionable authority on kringles.

In the late 1980s the focus of our research has moved to other fields, therefore our work on kringles and our collaboration with Miguel, temporarily, has ended in 1988. We have, however, learned an important lesson from our studies on kringles: the conservation of residues in different, non-orthologous kringles reflects their relative importance for the folding autonomy of kringle fold [44]. In this respect, it was noteworthy that the most highly conserved Trp25, Leucine 45 and Trp61 residues of kringles interact to form the core of the kringle-fold [16]. In other words, since the acceptance of mutations in a fold family depends on the role and importance of the affected residues in the protein fold, the pattern of conserved residues, variable sequences, regions that tolerate gap events etc. are characteristic of a protein fold. Accordingly, ‘consensus sequences’ incorporating these features may be used to decide whether a sequence has the features typical of the given protein fold, therefore they may be used to detect distant homologies [45]. The application of this principle allowed us to detect numerous “surprising” homologies, some of which were relevant for both fibronectin and the proteases of the fibrinolytic system. For example, we have demonstrated that the type I repeats (finger domains) of fibronectin are homologous with a domain of tissue-type plasminogen activator [46], whereas the type II repeats present in the gelatin-binding region of fibronectin are homologous with the kringle domains of proteases [47].

The latter finding has led us to initiate a new round of collaboration with Miguel’s group, this time on fibronectin type II repeats. Initially, the primary goal of this collaboration was to explore whether the distant homology of type II units and kringles is supported by their structural and functional similarities. In the first part of our work, we have studied a type II domain of the collagen binding bovine seminal fluid protein PDC-109, resulting in the first structure of a type II domain [48–50].

Our project on type II domains, however, gained additional interest when it turned out that gelatinases also contain type II domains. These metalloproteases play a key role in matrix remodeling, degradation of basement membranes and contribute significantly to the metastatic potential of tumor cells; they appeared promising targets for tumor therapy. Significantly, the three tandem type II domains of gelatinase A were shown to be responsible for the high affinity of the enzyme for collagen [51].

The NMR spectroscopic studies of Miguel on the various type II domains of gelatinase A [52–58] have provided important insight into the structure and function of these collagen-binding modules. These studies confirmed that kringle modules and fibronectin type II modules are related both in structure and function. This conclusion is now generally accepted; according to SCOP classification (http://scop.mrc-lmb.cam.ac.uk), kringle modules and fibronectin type II modules represent two families of the kringle-like superfamily.

Although our collaboration with Miguel Llinás was primarily through exchange of research materials via mail, exchanging ideas via email, we had regular personal contacts at the biannual meetings of the International Society for Fibrinolysis & Proteolysis (that has a kringle image in its logo) or at the various Plasminogen Activator workshops. I enjoyed his company as he had a good sense of humor, appreciated the pleasant aspects of life and had a broad interest in culture, history, music. He was an admirer of Bartók so I am glad that when he visited us in Hungary, I could show him the Béla Bartók Memorial House to get an impression about the life of this genius.

With Miguel’s passing, science has lost a dedicated scientist and I have lost one of the best friends I acquainted with during the heydays of Plasminogen Activation research.
